# Cor Triatriatum Dexter as an Incidental Finding: Role of Two-Dimensional Transthoracic Echocardiography

**DOI:** 10.7759/cureus.5683

**Published:** 2019-09-17

**Authors:** Bikramjit S Bindra, Zeel Patel, Neel Patel, Khushal V Choudhary, Vinod Patel

**Affiliations:** 1 Internal Medicine, Government Medical College and Hospital, Chandigarh, IND; 2 Internal Medicine, Ahmedabad Municipal Corporation Medical Education Trust Medical College, Ahmedabad, IND; 3 Internal Medicine, Interfaith Medical Center, Brooklyn, USA; 4 Internal Medicine, Roger Williams Medical Center, Providence, USA; 5 Cardiology, Mount Sinai Hospital, New-York City, USA

**Keywords:** ctd, cor triatriatum dexter

## Abstract

Cor triatriatum dexter (CTD) is a rare congenital cardiac anomaly in which a membranous structure divides the right atrium (RA) into two chambers. Persistence of the right valve of the sinus venosus, which usually regresses as a part of normal embryological development, is responsible for membranous partition. There is a high incidence of right-sided congenital abnormalities of the heart associated with this condition. Clinical manifestations vary depending on the degree of partitioning or septation of the RA. We present a case of CTD discovered as an incidental finding during transthoracic echocardiography and further discuss the role of two-dimensional echocardiography as a noninvasive diagnostic tool.

## Introduction

Cor triatriatum dexter (CTD), in which the right atrium (RA) is split into two chambers by a membrane, is an uncommon congenital abnormality with an estimated reported incidence of 0.1% of all congenital cardiac malformations. It results from persistence of the right valve of the sinus venosus [1]. The membrane can vary from a simple muscle bar to a fenestrated membrane or to a Chiari's network. The condition is associated with right-sided heart abnormalities, such as hypoplasia or atresia of the tricuspid and/or pulmonary artery orifice [2]. The extent of obstruction offered by the membrane and the right-sided heart abnormalities dictate symptomology [3].

## Case presentation

**Figure 1:**
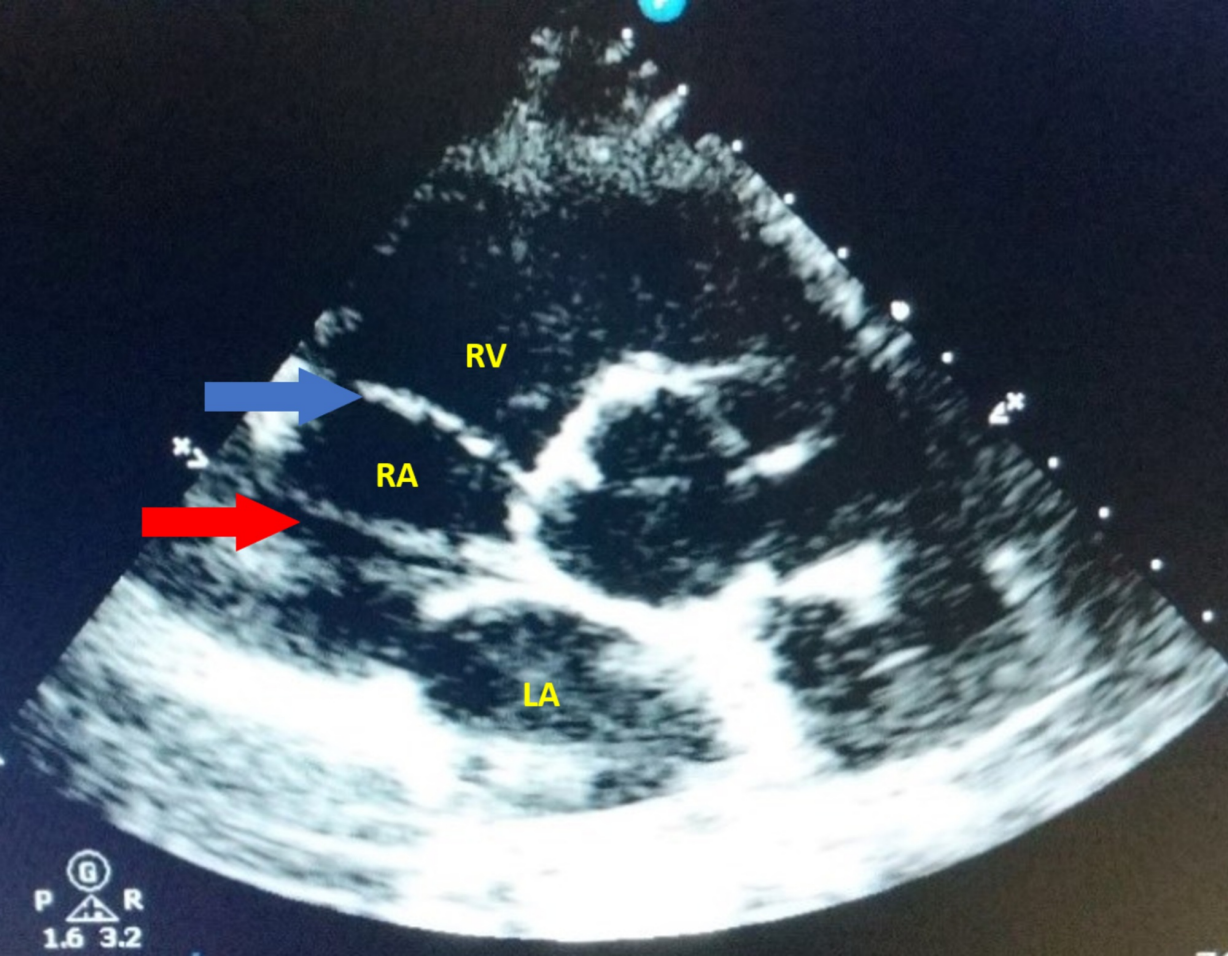
Transthoracic echocardiogram (TTE). Parasternal short-axis view showing a membranous structure (CTD) within the right atrial cavity (red arrow) and tricuspid valve (blue arrow). Abbreviations: LA, left atrium; RA, right atrium; RV, right ventricle

**Figure 2:**
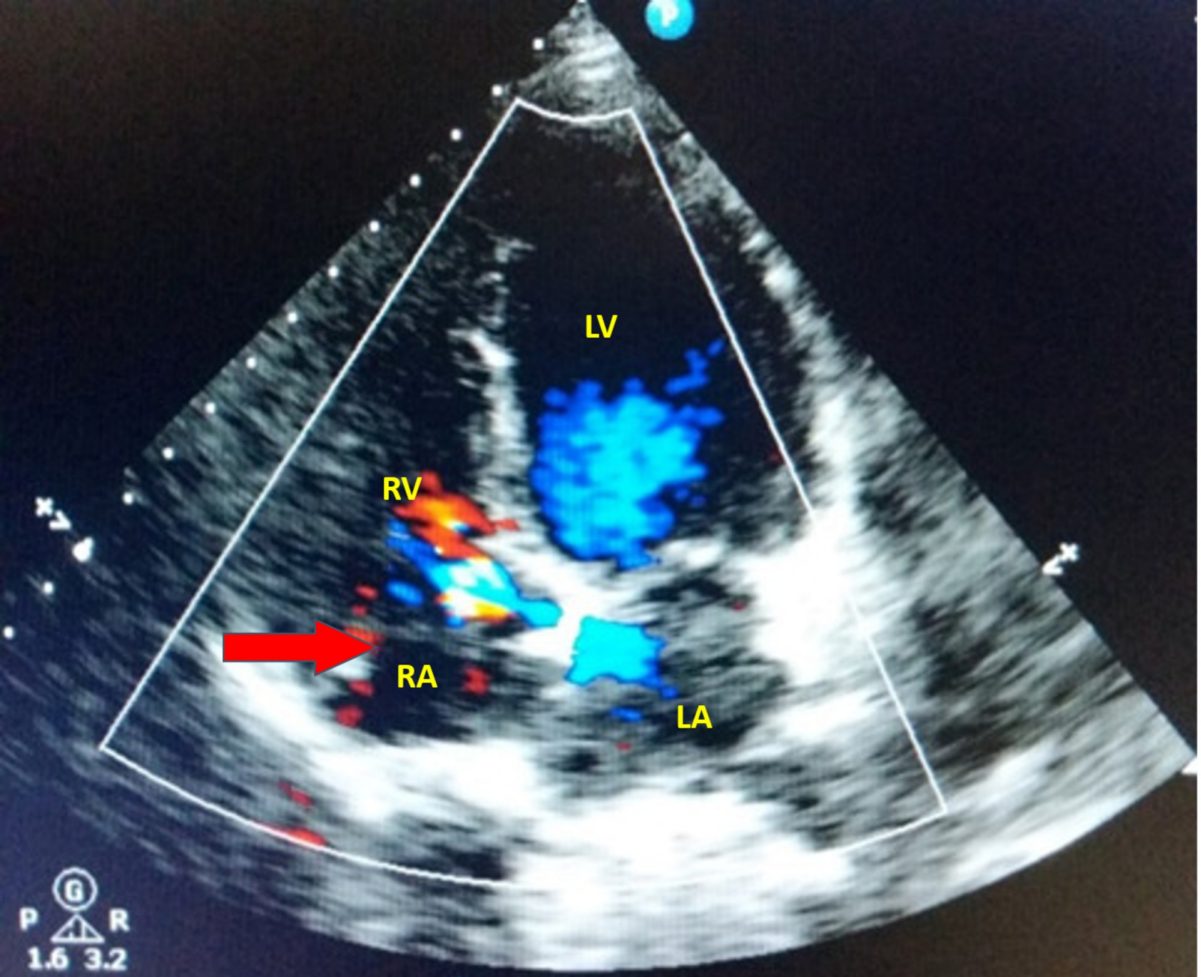
Color Doppler four-chamber view showing a membranous structure (CTD) within the right atrial cavity (red arrow). Abbreviations: LA, left atrium; LV, left ventricle; RA, right atrium; RV, right ventricle

A 38-year-old woman presented to an urgent care with chest pain. Her past medical history was significant for multiple similar episodes of chest discomfort and an episode of Lyme disease in her early twenties. Her cardiovascular examination was significant for a grade 2/6 early diastolic murmur over the left sternal area. A 12-lead electrocardiogram revealed Q-wave changes in V1/V2, suggestive of an old myocardial infarct involving the septal region. On two-dimensional (2D) transthoracic echocardiography (TTE) examination, a mobile membranous structure was visualized within the RA along with mild tricuspid and aortic valve regurgitation (Figure 1). The rest of the echocardiographic indices, including the ejection fraction, were within normal limits. Color flow Doppler did not reveal any obstruction to RA flow (Figure 2). A treadmill stress test performed to detect inducible cardiac ischemia showed no evidence for significant ischemia. As the echocardiographic indices were normal, and the stress test was negative, no medical intervention was provided, and the patient was discharged with directions to follow-up after one year.

 

 

## Discussion

CTD is an extremely rare congenital heart condition characterized by a membrane that divides the RA into an upstream chamber and a downstream chamber. The upstream chamber receives venous blood from the caval system and the coronary sinus, while the downstream chamber incorporates the tricuspid valve and right atrial appendage [4]. This membrane is a reminiscent structure formed due to the persistence of the right valve of the sinus venosus, an early embryonic structure that usually recedes between the ninth and the fifteenth week of gestation [1,5].

Clinical presentation of the patient varies, depending on the degree of partitioning or septation of the RA and the amount of blood flow across that membrane. When blood flow is adequate, the condition can be completely asymptomatic, in which case it is detected incidentally during routine echocardiography or surgery to correct other cardiac abnormalities. In contrast, an impeded flow can cause increased central venous pressure and severe right-sided heart failure [3]. Episodes of syncope, paradoxical systemic emboli, and supraventricular arrhythmias, such as atrial flutter, have also been reported in the literature [6,7].

CTD may be confused with a large, higher insertion-type Eustachian valve of inferior vena cava, a structure formed by the proximal portion of the same reminiscent membrane that gives rise to CTD. On an echocardiogram, the membrane in CTD can usually be visualized running from the inferior to the superior vena cava. However, the Eustachian valve originates from the margin of the inferior vena cava and may show rapid movement within the RA cavity, making it easy to be identified on cross-sectional echocardiographic examination [4,5,7,8].

CTD often occurs in combination with other right-sided structural cardiac defects, such as pulmonary artery stenosis or atresia, tricuspid valve abnormality, atrial septal defect, and Ebstein’s anomaly [3]. The occurrence of these defects in the right side of the heart could be explained by the reduced blood flow into the right ventricle due to the obstruction caused by the persistence of a wide right venous valve [9-11].

Diagnosing CTD can be difficult. Transthoracic or transesophageal echocardiography and three-dimensional echocardiography have been used to diagnose the condition [12-15]. Even though the latter is a more effective diagnostic modality, the lack of availability is often a limiting factor [16]. Some cases of the use of nuclear magnetic resonance have also been reported [17].

Although CTD is typically diagnosed in young patients, it can also present late in life in low acuity cases [18-20]. In the case we have discussed, it was an incidental finding.

Asymptomatic patients usually do not require any intervention. If the patient is undergoing some other surgical procedure, then resection of CTD can be clubbed along with the original procedure. In symptomatic patients, it is recommended that the membrane be surgically resected. Alternatives to surgical treatment like the percutaneous catheter-based disruption of the membrane have also been suggested [4].

Although CTD is extremely rare, its diagnosis should be considered in patients presenting with symptoms of right-sided heart failure, because it can be easily corrected by surgical excision or percutaneous approach.

## Conclusions

Routine echocardiographic assessment can sometimes reveal asymptomatic rare congenital anomalies of the heart, such as CTD. We have reported a case with acute chest discomfort where 2D TTE, performed as a part of routine cardiac assessment, revealed CTD as an incidental finding. The aim of this case report is to develop awareness regarding CTD. Being an extremely rare entity, not many physicians get a chance to encounter it during their practice.
